# Associations between serum bilirubin levels and essential trace elements status in an adult population

**DOI:** 10.18632/oncotarget.18351

**Published:** 2017-06-02

**Authors:** You-Fan Peng, Ye-Sheng Wei

**Affiliations:** ^1^ Department of Laboratory Medicine, Affiliated Hospital of Youjiang Medical University for Nationalities, Baise City, China

**Keywords:** serum bilirubin, serum iron, serum copper, serum zinc

## Abstract

**Objective:**

This paper aims to evaluate the relations between serum bilirubin and essential trace elements in an adult population.

**Results:**

Demographic and clinical data were stratified according to the median of serum bilirubin concentrations (50th percentiles). There were statistical differences in regarding with age, body mass index, white blood count, hemoglobin, mean corpuscular hemoglobin, alanine aminotransferase, creatinine, high-sensitivity C-reactive protein, iron, zinc and copper. Studying the correlation of serum bilirubin levels with iron, zinc, copper and high-sensitivity C-reactive protein, we found positive correlations for iron and zinc, and negative correlations for high-sensitivity C-reactive protein and copper in whole participants. Similar results of correlation analysis were repeated when the further analyses were performed separately for subjects with high and low serum bilirubin concentrations. Similar results were also observed in gender-based stratified analysis. Multiple linear regression analysis revealed that serum bilirubin levels were independently correlated with serum iron, zinc and copper.

**Materials and Methods:**

The cross-sectional study involved 264 healthy subjects.

**Conclusions:**

The current study demonstrated that serum bilirubin within the reference range is correlated with iron, zinc and copper in an adult population, regardless of potential confounders.

## INTRODUCTION

Iron, copper and zinc are essential trace elements in human body [1]. Previous studies showed that inflammation was a main factor to induce changes in zinc, iron and copper concentrations [2–3]. Indeed, low serum levels of iron have been found to be negative correlated with some traditional inflammatory markers such as high-sensitivity C-reactive protein (hs-CRP), tumor necrosis factor (TNF) and adiponectin [4], and inflammatory cytokines can decrease serum iron concentrations with no evidence of iron deficiency [5]. There was an association between serum zinc and inflammation in community population [6]. Tsuboi et al. [7] have suggested that serum zinc was correlated with higher hs-CRP in elderly women. Growing evidences have indicated that serum copper is related to inflammation in hospitalized patients and healthy volunteers [8–9]. Obviously, serum iron, copper and zinc levels may be associated with low-grade inflammatory conditions in human body [10].

Up to now, some serological indices such as serum bilirubin have been focused on diseases associated with inflammation. As the end product of heme catabolism in the systemic circulation, bilirubin has anti-inflammatory and anti-oxidative properties *in vitro* and *in vivo* [11]. In fact, bilirubin has more powerful antioxidant features than other antioxidants such as catalase, tocophero and superoxide dismutase [11]. In the clinical laboratory, a negative correlation between serum bilirubin and C-reactive protein (CRP) has been suggested by Lippi G et al. [12]. Lower serum bilirubin levels have been demonstrated in patients with monoxide poisoning, pulmonary embolism and chronic kidney disease [13–15], which indicates the possible relationship of serum bilirubin with inflammation and oxidative stress. Unfortunately, the inflammation and oxidative stress may have effect on essential trace elements metabolism, even in healthy subjects, and no study has analyzed the associations of serum bilirubin concentrations with trace elements in healthy individuals. Thus, this paper aims to evaluate the relations between serum bilirubin and iron, copper and zinc in adult population.

## RESULTS

### Baseline characteristics

Demographic and clinical data were stratified according to the median of serum bilirubin concentrations (50th percentiles), the median was 11.6 μmol/L for serum bilirubin levels. Table 1 summarizes the demographic and clinical features of the two groups. Subjects with high serum bilirubin levels had higher age, hemoglobin, MCH, ALT, Cr, iron, zinc values than subjects with low serum bilirubin concentrations. In contrast, body mass index, WBC, copper and hs-CRP decreased from the low to the high serum bilirubin concentrations. Other variables were not different between the two groups.

**Table 1:** Baseline characteristics of all subjects according to the median of serum bilirubin concentrations (50th percentiles)

	≤ 11.6	> 11.6	
	*N* = 129	*N* = 135	*P*-value
Gender (F/M)	57/72	62/73	0.776
Age (yr)	28.4 ± 18.75	41.2 ± 15.58	< 0.001
Body mass index (kg/m^2^)	23.4 ± 2.42	22.8 ± 2.63	0.029
White blood count (10^9^/L)	6.7 ± 1.44	6.3 ± 1.47	0.016
Hemoglobin (g/L)	128.8 ± 12.80	134.3 ± 12.31	< 0.001
Mean corpuscular hemoglobin (pg)	27.2 ± 2.98	28.5 ± 3.07	< 0.001
Mean corpuscular hemoglobin concentration (g/L)	326.5 ± 10.64	326.8 ± 27.54	0.902
Alanine aminotransferase (U/L)	17.5 ± 8.86	19.6 ± 8.40	0.047
Aspartate aminotransferase (U/L)	22.3 ± 6.20	21.9 ± 5.54	0.669
Creatinine (umol/L)	58.1 ± 17.60	70.1 ± 12.94	< 0.001
Albumin (g/L)	44.7 ± 3.53	44.6 ± 3.26	0.850
Globulin (g/L)	22.1 ± 4.09	22.8 ± 3.84	0.180
High-sensitivity C-reactive protein (mg/L)	1.4 ± 1.03	1.0 ± 0.90	0.002
Serum iron (μmol/L)	11.9 ± 1.88	15.7 ± 5.64	< 0.001
Serum copper (μmol/L)	12.7 ± 4.51	10.0 ± 3.22	< 0.001
Serum zinc (μmol/L)	10.9 ± 1.88	12.0 ± 2.64	< 0.001

### The correlation analysis between serum bilirubin and trace elements

Serum bilirubin was found to be positive correlated with age, hemoglobin, MCH, MCHC, ALT and Cr, and negative correlated with body mass index, WBC and hs-CRP in all participants. Studying the correlation of serum bilirubin levels with iron, zinc and copper, we found positive correlations for iron and zinc, and negative correlation for copper in whole participants. Similar results were also observed in gender-based stratified analysis. The further analyses were performed separately for individuals with high and low serum bilirubin concentrations. The correlation analysis showed that serum bilirubin was positive correlated with iron and zinc, and was negative correlated with hs-CRP and copper in subjects with high serum bilirubin levels, similarly, serum bilirubin was positive correlated with age, hemoglobin, MCH, MCHC, ALT, Cr, iron and zinc, and was negative correlated with hs-CRP and copper in subjects with low serum bilirubin levels, as shown in Table 2.

**Table 2:** Correlations between serum bilirubin and demographic and laboratory parameters

	All subjects		Subjects with high serum bilirubin		Subjects with low serum bilirubin	
	*r*	*P*-value	*r*	*P*-value	*r*	*P*-value
Age	0.377	< 0.001	0.001	0.992	0.305	< 0.001
Body mass index	−0.157	0.011	−0.024	0.785	−0.118	0.171
White blood count	−0.133	0.031	0.113	0.193	−0.051	0.567
Hemoglobin	0.285	< 0.001	0.082	0.346	0.230	0.009
Mean corpuscular hemoglobin	0.350	< 0.001	0.038	0.662	0.314	< 0.001
Mean corpuscular hemoglobin concentration	0.171	0.005	0.064	0.460	0.207	0.019
Alanine aminotransferase	0.215	< 0.001	0.029	0.741	0.268	0.002
Aspartate aminotransferase	0.034	0.587	0.029	0.738	0.108	0.224
Creatinine	0.370	< 0.001	0.067	0.440	0.218	0.013
Albumin	0.011	0.865	−0.011	0.906	0.004	0.961
Globulin	0.087	0.158	0.083	0.348	−0.002	0.983
High-sensitivity C-reactive protein	−0.311	< 0.001	−0.187	0.030	−0.325	< 0.001
Serum iron	0.452	< 0.001	0.275	0.001	0.394	< 0.001
Serum copper	−0.475	< 0.001	−0.266	0.002	−0.485	< 0.001
Serum zinc	0.311	< 0.001	0.180	0.037	0.335	< 0.001

r: correlation coefficient.

Note: High corresponds to individuals with bilirubin levels higher than 11.6 μmol/L.

### Serum bilirubin and trace elements in multiple linear regression analysis

Multiple linear regression analysis revealed that serum bilirubin levels were independently correlated with serum iron, zinc and copper (beta = 0.276, *P* < 0.001; beta = 0.312, *P* < 0.001; beta = −0.304, *P* < 0.001), when gender, age, body mass index, WBC, hemoglobin, MCH, ALT, AST, Cr, hs-CRP, iron, zinc and cooper were considered as independent variables, and serum bilirubin were served as dependent variables in multiple linear regression analysis (Table 3).

**Table 3:** Potential factors associated with serum bilirubin in multiple linear regression

	Unstandardized cofficients		Standardized cofficients	*t*	*P*-value
	B	Std Error	Beta		
Age	0.054	0.012	0.215	4.691	< 0.001
Body mass index	−0.197	0.080	−0.108	−2.464	0.014
Mean corpuscular hemoglobin	0.148	0.070	0.099	2.110	0.036
Serum iron	0.236	0.041	0.276	5.816	< 0.001
Serum copper	−0.343	0.053	−0.304	−6.517	< 0.001
Serum zinc	0.613	0.090	0.312	6.790	< 0.001

B: regression coefficient; Std Error: standard error.

## DISCUSSION

As far as we know, this is the first study to investigate the associations between serum bilirubin and essential trace elements in an adult population. The major findings of the current study are that serum bilirubin is positive correlated with iron and zinc, and negative correlated with copper in an adult population, further, serum bilirubin is independently correlated with serum iron, zinc and copper in multiple linear regression analysis.

Serum bilirubin has been used as an outstanding diagnostic marker in liver diseases such as acute hepatitis, liver cirrhosis and autoimmune hepatitis [16]. Previous clinical studies have demonstrated that serum bilirubin concentrations are related with chronic obstructive pulmonary disease, migraine and cancer [17–19]. Kawamoto et al. [20] found that serum bilirubin was useful as an underlying risk marker for kidney function. Moreover, a reverse relationship between serum bilirubin and carotid atherosclerosis has been suggested in elderly population [21], and serum bilirubin concentrations are correlated with disease activity in patients with rheumatoid arthritis and polymyositis [22–23]. In other oxidative stress-mediated diseases, lower serum bilirubin levels have been also reported to be inverse associated with Takayasu Arteritis, coronary atherosclerosis and myocardial infarction [24–26]. The present results found that the physiological serum bilirubin levels were tightly correlated with essential trace elements such as iron, zinc and copper in an adult population. Although the mechanisms in regarding with the correlations between serum bilirubin and these trace elements have not been clear, however, inflammation may be a bridge to explain the relations in the study population.

Inflammation has an important effect on nutrient status, particularly for essential trace elements [27]. Some studies found the associations between CRP and serum iron, zinc and copper [28–29]. There is evidence that zinc presents strong anti-oxidative and anti-inflammatory features [30]. Serum zinc concentrations have been found to be correlated with inflammatory markers, including interleukin-6 (IL-6), TNF, and CRP, and serum zinc decreased these inflammatory cytokines by the downregulation of NF-kB activation [31–32]. In addition, low serum zinc and high serum copper concentrations have been demonstrated to be associated with inflammatory factors in patients with cardiovascular disease [33]. Further, copper has been regarded as an inflammatory biomarker with synthesis and secretion during inflammation [34]. Inflammation has also important impact on iron metabolism [5]. In conclusion, inflammation may increase serum copper concentrations, whilst decrease serum zinc and iron levels, even in healthy subjects. Bilirubin is a powerful endogenous anti-oxidant that scavenges peroxyl radicals and inhibits oxidative stress, and bilirubin has anti-inflammatory and anti-oxidative and immunosuppressive characteristics [35]. It is suggested that serum bilirubin concentrations are directly correlated with serum antioxidant capacity [36]. Importantly, bilirubin has been reported to be a protector in inflammatory processes in vasculature [37]. We speculate that serum bilirubin may be destroyed and consumed against inflammation in human body. Interesting, in agree with the possibility, a reverse correlation between serum bilirubin and hs-CRP was observed in our study. Therefore, the relations of serum bilirubin with iron, zinc and copper should be attributed to systemic low-grade inflammation. On the other hand, in serum, copper and iron are mostly bound to ceruloplasmin and transferrin respectively, and zinc is bound to albumin and a-macroglobulin. In fact, these proteins related with trace elements are associated with acute phase proteins (APPs), and CRP is positive correlated with APPs. Thus, the elevation of the bilirubin levels is related with low serum CRP levels due to its anti-inflammatory response. In a healthy population, relatively high levels of bilirubin are related with low CRP levels, so that it can explain the correlation observed with the copper, iron and zinc levels under this condition.

Several limitations should be acknowledged. First, our study was limited by a small sample, resulting in a low statistical power. Second, the information on food intake was not available, which may have an effect on serum iron, copper and zinc in this cross-sectional study, even though the albumin, globulin and body mass index were used as complementary data for the general nutritional status in our study. Third, we have no the physiology information in the study population, such as menstruation in females. Fourth, more accurate measurement methods for serum copper, iron and zinc were needed in the present study. Fifth, because the study only included healthy subjects, the extrapolation of results may be limited in hospitalized patients. Finally, the detailed information on drugs and supplements was not obtained in all subjects, which may contain trace elements. The current study demonstrated that serum bilirubin within the reference range is correlated with iron, zinc and copper concentrations in an adult population, regardless of potential confounders. However, further studies were needed in different settings with a larger sample size.

## MATERIALS AND METHODS

### Study population

The cross-sectional study involved 264 healthy subjects from the Affiliated Hospital of Youjiang Medical University for Nationalities between November 2015 and August 2016, and all subjects underwent laboratory and physical examinations. Our study included healthy subjects without apparent diseases. Following participants with clinical findings are ineligible: diabetes, hypertension, cardiovascular disease, dyslipidemia, renal or liver dysfunction, acute or chronic infectious disease, inflammatory disease, anemia, malignant tumor, smoking, alcohol consumption, mental disorders and pregnancy. Our study was approved by the Affiliated Hospital of Youjiang Medical University for Nationalities Institutional Review Board.

### Clinical and laboratory variables

Fasting venous blood was used for all participants. All laboratory indexes were measured within 2 hours. White blood count (WBC), hemoglobin, mean corpuscular hemoglobin (MCH) and mean corpuscular hemoglobin concentration (MCHC) were measured by automated hematology analyzer (Sysmex XN2000, Japan). Serum bilirubin, alanine aminotransferase (ALT), aspartate aminotransferase (AST), creatinine (Cr), albumin(ALB), hs-CRP, iron, copper and zinc were measured by automatic biochemical analyzer (Hitachi7600, Japan). The body mass index was calculated as an individual's weight in kilograms divided by the square of height in meters.

### Determination of laboratory variables

The kits of serum bilirubin, ALT, AST, Cr, ALB, hs-CRP, iron, copper and zinc were purchased from Maker Biotechnology co., LTD. We used oxidizing method (vanadate as oxidizing reagent), alanine substrate method, 2,4-dinitrobenzene hydrazine method, sarcosine oxidase method and bromocresol green method to measure serum bilirubin, ALT, AST, Cr, ALB levels, respectively. The serum concentrations of hs-CRP were measured by immunoturbidimetry, and serum iron, copper and zinc were assessed by colorimetric method. All tests were performed in the identical laboratory.

### Statistical analysis

Data analyses were performed by SPSS 16.0 software (SPSS Inc., Chicago, IL, USA). The normal distribution for continuous variables was tested by Kolmogorov–Smirnov test. Intergroup comparisons between the two groups were assumed by student's *t*-test, Mann-Whitney *U* test and Chi-square test. Correlations between serum bilirubin and other variables were calculated by Spearman's test. Multiple linear regression analysis was used to identify the correlations between serum bilirubin level and iron, copper and zinc in the study population. *P* < 0.05 was used as the criterion of statistical significance.
